# Tracking Infection and Genetic Divergence of Methicillin-Resistant *Staphylococcus aureus* at Pets, Pet Owners, and Environment Interface

**DOI:** 10.3389/fvets.2022.900480

**Published:** 2022-06-02

**Authors:** Muhammad Shoaib, Amjad Islam Aqib, Muhammad Muddassir Ali, Muhammad Ijaz, Huma Sattar, Awais Ghaffar, Muhammad Sajid Hasni, Zeeshan Ahmad Bhutta, Khurram Ashfaq, Muhammad Fakhar-e-Alam Kulyar, Wanxia Pu

**Affiliations:** ^1^Key Laboratory of New Animal Drug Project, Gansu Province, Key Laboratory of Veterinary Pharmaceutical Development, Ministry of Agriculture and Rural Affairs, Lanzhou Institute of Husbandry and Pharmaceutical Sciences of CAAS, Lanzhou, China; ^2^Institute of Microbiology, Faculty of Veterinary Science, University of Agriculture Faisalabad, Faisalabad, Pakistan; ^3^Department of Medicine, Cholistan University of Veterinary and Animal Sciences, Bahawalpur, Pakistan; ^4^Institute of Biochemistry and Biotechnology, University of Veterinary and Animal Sciences, Lahore, Pakistan; ^5^Department of Veterinary Medicine, University of Veterinary and Animal Sciences, Lahore, Pakistan; ^6^Institute of Molecular Biology and Biotechnology, The University of Lahore, Lahore, Pakistan; ^7^Department of Clinical Sciences, KBCMA, College of Veterinary and Animal Sciences, Narowal, University of Veterinary and Animal Sciences, Lahore, Pakistan; ^8^Directorate General Farms and Feed Resources, Livestock and Dairy Development Department, Quetta, Pakistan; ^9^Laboratory of Biochemistry and Immunology, College of Veterinary Medicine, Chungbuk National University, Cheongju, South Korea; ^10^Department of Clinical Medicine and Surgery, University of Agriculture, Faisalabad, Pakistan; ^11^College of Veterinary Medicine, Huazhong Agricultural University, Wuhan, China

**Keywords:** *S. aureus*, mecA gene, pets, MRSA, phylogenetic analysis, pet owners

## Abstract

*Staphylococcus aureus* (*S. aureus*) has become a leading animal and public health pathogen that keeps on transferring from one host to other, giving rise to newer strains by genetic shifts. The current study was designed to investigate the epidemiology and genetic relatedness of *mecA* gene in *S. aureus* isolated from pets, immediate individuals in contact with pets, and veterinary clinic environments. A total of *n* = 300 samples were collected from different veterinary hospitals in Pakistan using convenience sampling. The collected samples were subjected to microbiological and biochemical examination for the isolation of *S. aureus*. Methicillin resistance was investigated by both phenotypically using oxacillin disk diffusion assay and by genotypically targeting *mecA* gene by PCR. PCR amplicons were subjected for sequencing by Sanger method of sequencing, which were subsequently submitted to NCBI GenBank under the accession numbers MT874770, MT874771, and MT874772. Sequence evolutionary analysis and *mecA* gene characterization was done using various bioinformatics tools. Overall, 33.66% *mecA* genes harboring *S. aureus* strains were isolated from all sources (33.33% from pets, 46.0% from surrounding, and 28.0% from immediate contact individuals). The bioinformatics analysis noted that one SNP was identified at position c.253C>A (Transvertion). The phylogenetic tree (two clades) of *S. aureus mecA revealed a possibility of inter-transmission of disease* between the environment and pets. Frequency of adenine and thymine nucleotide in motifs were found to be the same (0.334). Cytosine and guanine frequency were also the same (0.166). Threonine was replaced by asparagine (p.T84D) in each sample of cat, environment, and human. On the other hand, protein structures ofcat-1 and cat-2 proteins were found identical while cat-3, environmental, and human proteins shared identical structures. The study thus concludes rising circulation of methicillin-resistant *S. aureus* (MRSA) strains in animal-human-environment interfaces, forecasting the development of novel strains withmodified range of resistance.

**Graphical Abstract:**
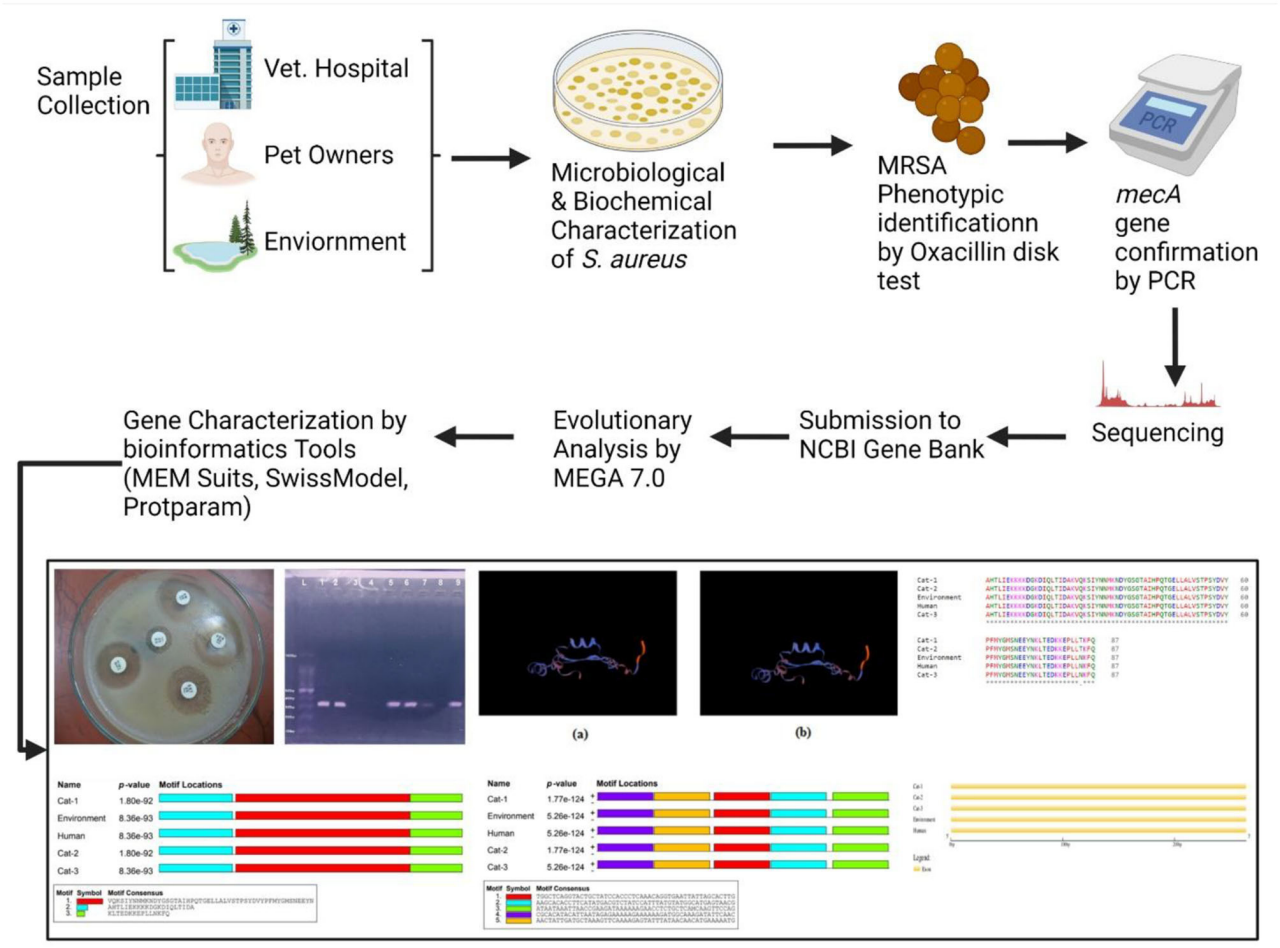
Representing the schematic diagram from sample collection to sequence analysis.

## Introduction

Over the lastdecade, with growing trends in pet or companion adoption, pedigree animals are imported for various purposes (shows, sports, pets, and breeding), and public awareness of animal welfare have raised concerns among veterinary researchers of Pakistan for treatment of animal diseases and/or common human and veterinary pathogens in Pakistan. *Staphylococcus aureus* (*S. aureus*) is a key pathogen that causes illnesses ranging from mild skin infections to life-threatening diseases in humans and animals, such as sepsis, pneumonia, endocarditis, deep rooted abscesses, and toxic shock syndrome. *Staphylococcus aureus* is an opportunistic pathogen that is capable of colonizing the skin, mucosal surface, gastrointestinal tract (GIT), urogenital tract, and respiratory tract (1). Within a couple of years, *S. aureus* has emerged to be a well-known pathogen due to its ability to develop resistance to commonly used antimicrobials and infect a significantly wider range of hosts (2–4). After horizontal gene transfer (HGT) and recombination, it becomes the storehouse of antibiotic resistance genes (ARG) and other virulence factor-encoding genes. The genomic divergence of *S. aureus* has led to the development of highly resistant strains that may cause serious problems with antibiotic treatment (5). Methicillin-resistant *S. aureus* (MRSA) that harbors the *mec A* gene has emerged to be a serious problem worldwide and is becoming more common in humans and animals. These strains are now recognized worldwide as livestock-associated (LA-MRSA), healthcare-associated (HA-MRSA), and community-associated (CA-MRSA) infections (6).

The transmission of *S. aureus* occurs either through direct human-to-human and/or human-to-animals contact with asymptomatic carriers (7). Until 2003, most of the identified MRSA clones belong to multi-locus sequence clones associated with human transmission and infection. The outbreak of the CC398 clone, considered as LA-MRSA clone, was found in farm animals and farm workers, indicating that certain MRSA strains are not strictly restricted to host species (8). A study conducted in the Netherlands found a high prevalence of MRSA in slaughtering pigs despite of reduction in antibiotic usage among pigs. Among the detected MRSA, 39% belonged to the ST398 complex (9). MRSA ST398 can cause infections in humans, suggesting that close contact with animals is an important risk factor for transfer of MRSA clones between human and animals (10).

Methicillin-resistant *S. aureus* harboring *mecA* gene is present within Staphylococcal Chromosomal Cassette mec (*SCCmec*) (11), and exhibits a decreased affinity to β-lactam and penicillin classes of antibiotics (12). *SCCmec* is a mobile element involved in the development of resistance to major antibiotics (e.g., β-lactams, macrolides, aminoglycosides, etc.). The phylogenetic patterns of the resistance genes encoded by *staphylococcus* SCCmec significantly differ from the phylogenetic patterns of the main genes, indicating that these genes are frequently switched between staphylococcal species. Due to the large population and the short replication time of bacteria, resistance develops within 2–4 years after the introduction of new antibiotics (13). Majority of isolated *S. aureus* strains have been identified using disk diffusion test. Further, *mec A* gene confirmation can done through PCR after preliminary identification by disk diffusion test (14).

Many reports of MRSA infections from animals to humans have been documented, but in Pakistan, there is lack of knowledge on MRSA transmission at the pet-human and environment cadre. The present study was planned to study the evolutionary phylogenetic analysis of *mecA* gene in *S. aureus* to analyze the distribution of *S. aureus-*causing infections within the same geographical environment within a certain period. We also discuss the phylogenetic placement of sequenced strains from Pakistan and worldwide by investigating the evolutionary histories of methicillin resistance in a phylogenetic framework.

## Materials and Methods

### Ethical Concern

A consent form was filled before taking samples by nasal swabs from nostrils of humans and animals in accordance with the standard ethical guidelines. The study was approved by departmental and faculty research committee and completion of study was notified *via* no.: CE/1701/2019.

### Collection of Samples

A total of 300 samples were collected including cats (*n* = 150), cat owners (*n* = 100), and environment (*n* = 50) from public and private veterinary hospitals located in Faisalabad, Punjab, Pakistan. During sampling, sterilized swabs were inserted into the nostril at a depth of approximately 1 cm before being rotated five times, while environmental samples were taken from the surface of instruments, tables, and animal keepings. Samples were transported in Amie's medium to the laboratory of Institute of Microbiology, University of Agriculture Faisalabad and maintained in a cold chain (4°C) for further investigation.

### Phenotypic Identification of MRSA

Each sample (10 μl) was disseminated on blood agar and incubated at 37°C for 24 h. These were then subsequently placed on Mannitol Salt Agar (MSA) for identification of *S. aureus* which was confirmed through culture characteristics, biochemical tests, and microscopic examination as recommended in the Bergey's Handbook of Determinative Bacteriology (15). Phenotypic identification of MRSA was carried out by oxacillin disk diffusion test in accordance with the recommendations of Clinical Laboratory Standard Institute (16).

### Molecular Detection and Sequencing of *MecA* Gene

The DNA of *S. aureus* isolates were extracted through WizPrep™ gDNA Mini Kit (Cell/Tissue; Wizbiosolutions Inc. South Korea) following the manufacturer's guidelines. For PCR amplification, a total of 20 μl reaction mixture containing 10 μl of master mix (GeneDireX, Inc. USA), 3 μl extracted DNA (50 ng/μl) that was used as template, 3 μl of DNA free water, and 2 μl (20 pmoL) of each forward and reverse primer of *mecA* gene (Table 1) was prepared. The thermocycler conditions were as follows: initial denaturation at 95°C for 5 min, followed by 35 cycles of final denaturation 95°C for 30 s, annealing at 58°C for 30 s, initial extension at 72°C for 30 s, and a final extension at 72°C for 10 min. The presence of amplicons was determined by gel electrophoresis of 10 μl of products in 1.5% agarose gel containing 10 μl ethidium bromide for staining of DNA at 200 amperes and 120 volts for 30 min. The stained gel was visualized under UV light. The amplicons were subjected for sequencing to a 1st BASE DNA sequencing company (Singapore) that uses Applied Biosystems™ BigDye™ Terminator v3.1 Cycle Sequencing Kit (Thermo Fisher Scientific, USA) using Sanger method of sequencing.

**Table 1:** Nucleotide sequence of the primers used for detection of *mecA* gene.

**Primer gene**	**Oligonucleotide sequence (5′-3′)**	**Amplicon size (bp)**	**Reference**
*mec A gene*	**F:** 5**′**-TGGCATTCGTGTCACAATCG-3**′**	310	(17)
	**R:** 5**′**-CTGGAACTTGTTGAGCAGAG-3**′**	310	

### Sequence Analysis

The *mecA* gene sequences from each source (cat, cat owner, and environment) was subjected to evolutionary analysis by MEGA 7.0 software. For this, the sequence was first analyzed through BLAST to get highly similar nucleotide sequences (*n* = 36) from NCBI nucleotide GenBank, and alignment was done through Clastal W software. The evolutionary history was accomplished by employing the Minimum Evolution (ME) methodology and replicating tree percentage, in which the associated taxa was clustered together in the bootstrap test (1,000 replicates). The distance of evolution was calculated in relation to the number of base substitutions at each site using the 2-parameter Kimura method. The initial phylogenetic tree was developed with *mecA* gene sequences from all sources by using neighbor-joining with a bootstrap value of 1,500, minimum evolution algorithm at 1.91 optimal tree branch length, and 0.1 evolutionary distance. The final phylogeny tree was constructed using a MEGA 7.0 software to investigate the evolution of *mecA* homologs.

Single nucleotide polymorphisms were identified in sequencing chromatograms using chromas software. Multiple Expectation maximizations for Motif Elicitation (MEME) Suit was used for the construction of nucleic acid and protein motifs. Gene structure display server software was used to construct the gene structure. Protein structures (3D) were predicted by Swiss model. Protparam was used to predict the proteins physical and chemical parameters.

### Submission of Sequence to NCBI GenBank

The *mecA* gene sequences analyzed by Sanger method of sequencing from all sources were subjected to submission under NCBI GenBank. After complete submission and evaluation, sequences were accepted for publication in their repository. Accession numbers were issued as identifiers for each submitted sequence, namely, MT874770 (Environment), MT874771 (pet-owner), and MT874772 (Cat).

### Analysis of Data

The data were analyzed using SPSS statistical computer software version 26, and sequence analyses was done using various online and offline bioinformatics tools.

## Results

**Figure 1:**
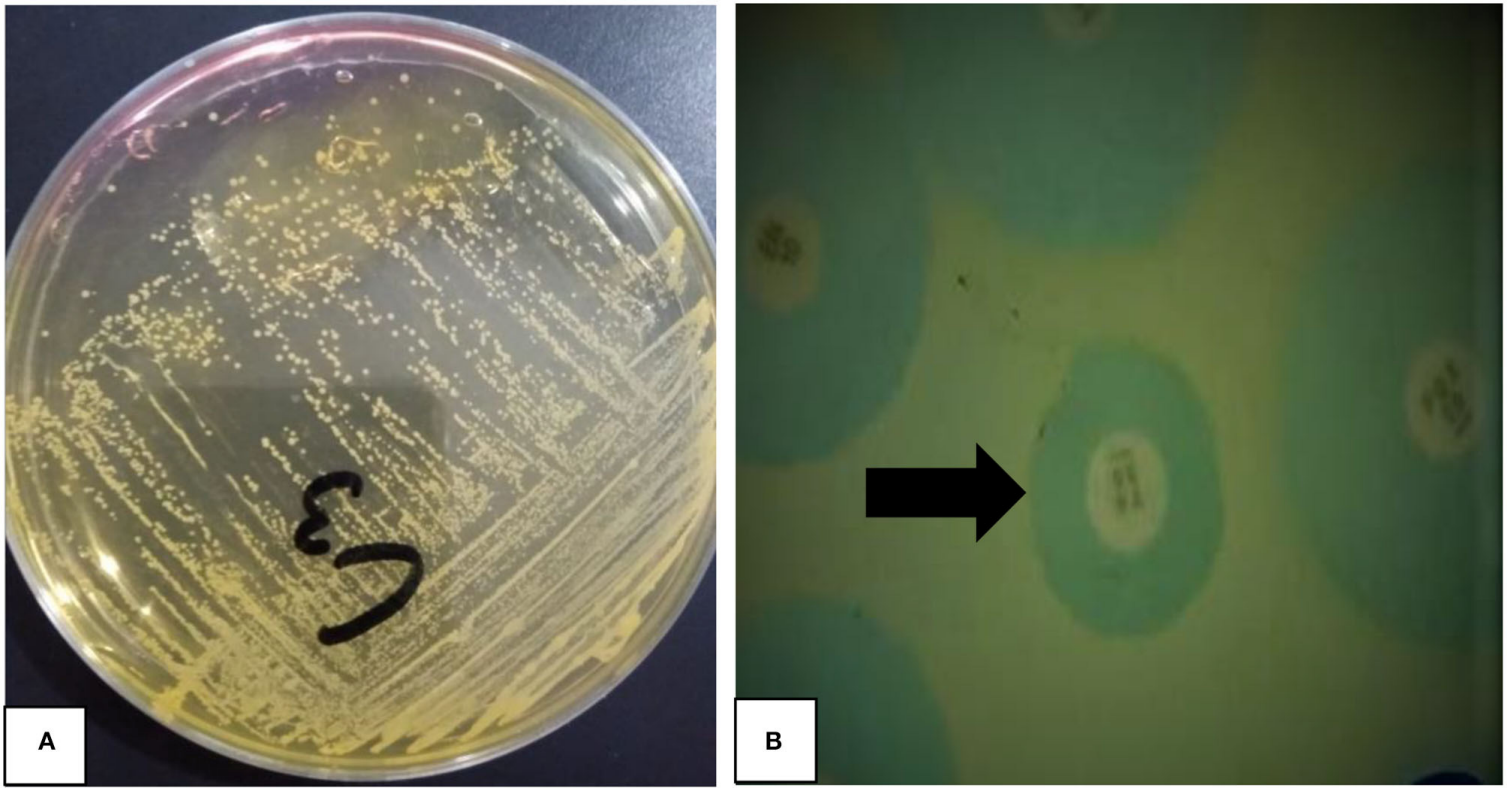
Phenotypic identification of *Staphylococcus aureus* and methicillin-resistant *S. aureus* (MRSA). **(A)** A Yellow color growth of *S. aureus* (pinpoint colonies) on mannitol salt agar, NB: C3 mentioned on plate is sample number tested **(B)** methicillin resistant *S. aureus* (MRSA) identified by oxacillin disc (black arrow) diffusion assay.

**Figure 2:**
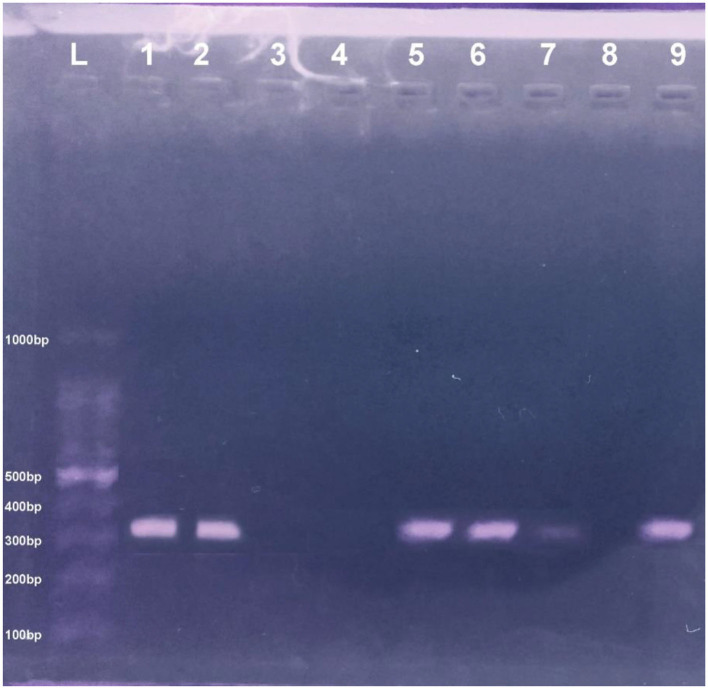
PCR pic of *mecA* gene at 310 bp. Lane L showing the ladder DNA, where Lane 8 and 9 are negative and positive control, respectively, and Lane 1–2 and 5–7 show *mecA* positive samples while Lane 3–4 express negative *mecA* isolates.

The present study found 101 (33.66%) *mecA* gene positive (MRSA) isolates from 300 pets (cats), individuals having direct contact with pet, and pet environment. The phenotypic identification of *S. aureus* and MRSA was done by MSA culture and oxacillin disk diffusion test, respectively (Figure 1). The *mecA* gene was identified by amplification through PCR, and *mecA* positive isolates from different sources were presented (Figure 2). Thirty-three-point-thirty-three percent (50/150) of *mecA* gene harboring *S. aureus* isolates were found in cats, 46.0% (23/50) from environment, which were twice to the isolates from pet owners [28.0% (28/100)]. In this study, there were no significant differences between *S. aureus* in animal, animal, and human interactions (*P* > 0.05), but a significant difference was noted in *mecA* gene prevalence among members (*P* < 0.05; Table 2).

**Table 2:** Infection status of methicillin resistance *Staphylococcus aureus* (*mecA* gene) isolates.

**Sample sources**	**Location/hospital name**	**Total sample**	Positive *S. aureus* (%)^*^	*mecA* gene positive (%)^**^
Cats	UAF Teaching Hospital Faisalabad, Pakistan	70	62 (88.57)	27 (38.57)
	Al-Huda Pet Clinic and Boarding Center Faisalabad, Pakistan	30	21 (70.0)	13 (43.33)
	Pet-Aid Clinic Faisalabad, Pakistan	50	37 (74.0)	10 (20.0)
	Total	150	120 (80.0)	50 (33.33)
Pet-Owner	UAF Teaching Hospital Faisalabad, Pakistan	40	28 (70.0)	12 (30.0)
	Al-Huda Pet Clinic and Boarding Center Faisalabad, Pakistan	30	17 (56.66)	9 (30.0)
	Pet-Aid Clinic Faisalabad, Pakistan	30	20 (66.66)	7 (23.33)
	Total	100	65 (65.0)	28 (28.0)
Environment	UAF Teaching Hospital Faisalabad, Pakistan	20	15 (75.0)	10 (50.0)
	Al-Huda Pet Clinic and Boarding Center Faisalabad, Pakistan	15	10 (66.66)	8 (53.33)
	Pet-Aid Clinic Faisalabad, Pakistan	15	10 (66.66)	5 (33.33)
	Total	50	35 (70.0)	23 (46.0)
**Overall total**		300	220 (73.33)	101 (33.66)

* *No significant difference (P > 0.05) was noted at Staphylococcus aureus cadre*.

** *Significant difference (P < 0.05) was noted at MRSA cadre among different sources*.

In present study, bacteriological and biochemical analysis of samples from cats, cat owners, and environments depicted the harboring of the *mecA* positive *S. aureus*. The conventional PCR results show the significant bands of *mecA* gene isolated from cats, cat owners, and environments (310 bp), as shown in Figure 2. The retrieved nucleotide sequences of targeted *mecA* genes were submitted to the NCBI GenBank with accession numbers MT874770 (Environment origin), MT874771 (pet-owner origin), and MT874772 (Cat origin).

**Figure 3:**
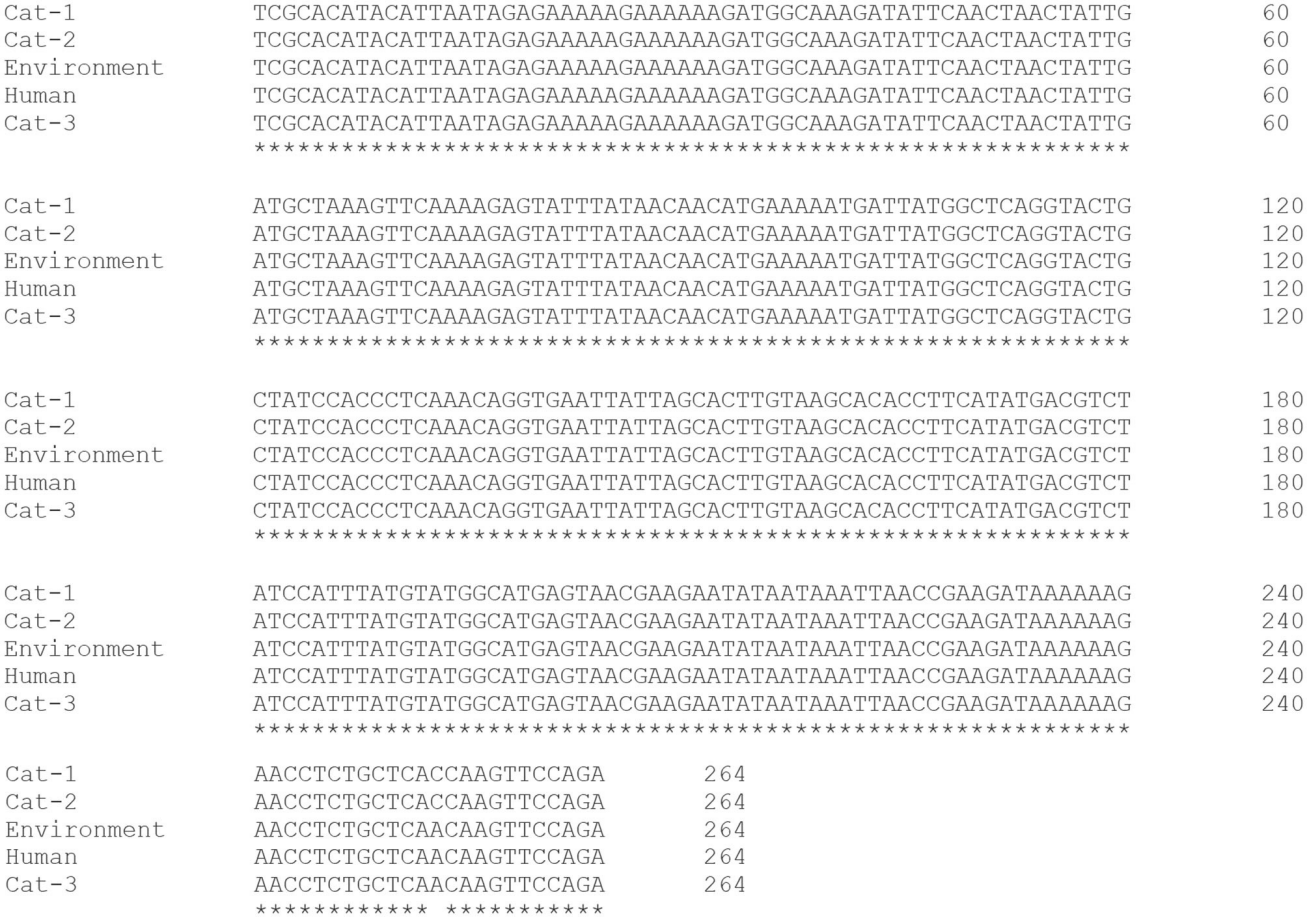
Alignment of *Staphylococcus aureus mecA* gene (Cat-1, Cat-2, Cat-3, Environmental, and Human sequence).

**Figure 4:**
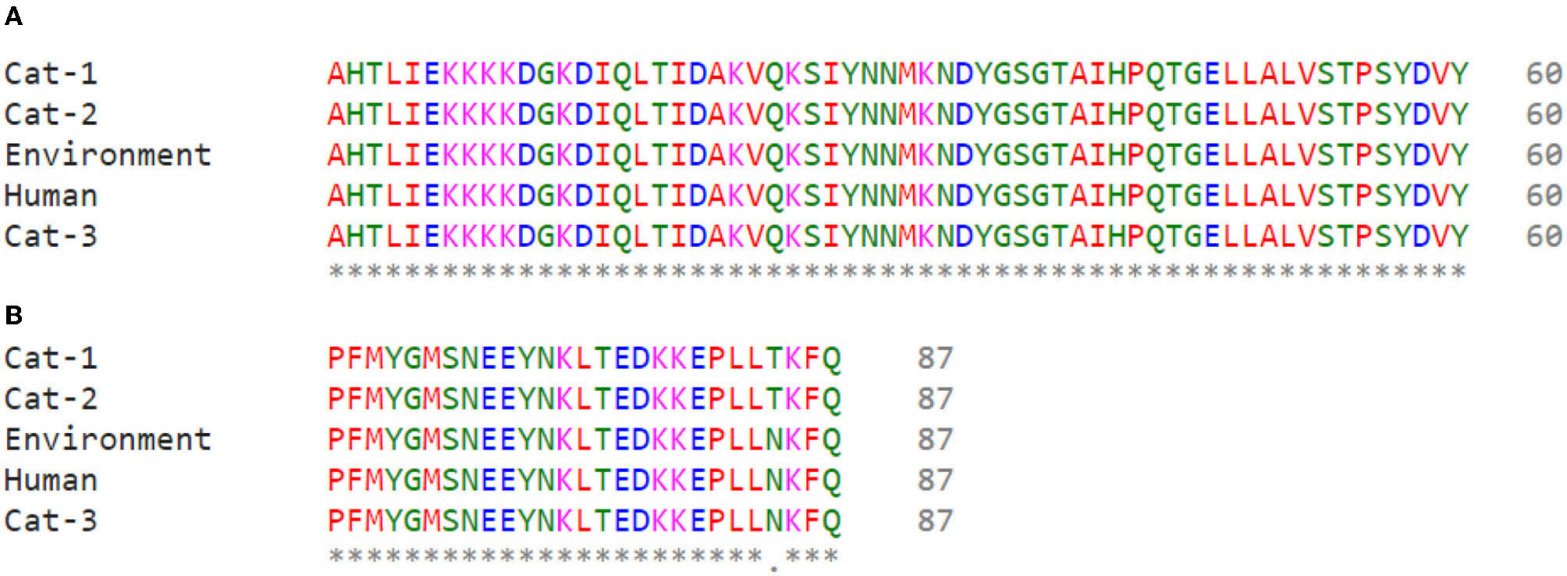
Alignment of Staphylococcus aureus mecA protein sequences **(A)** Cat-1 and Cat-2 **(B)** Environment, Human, and Cat-3.

**Figure 5:**
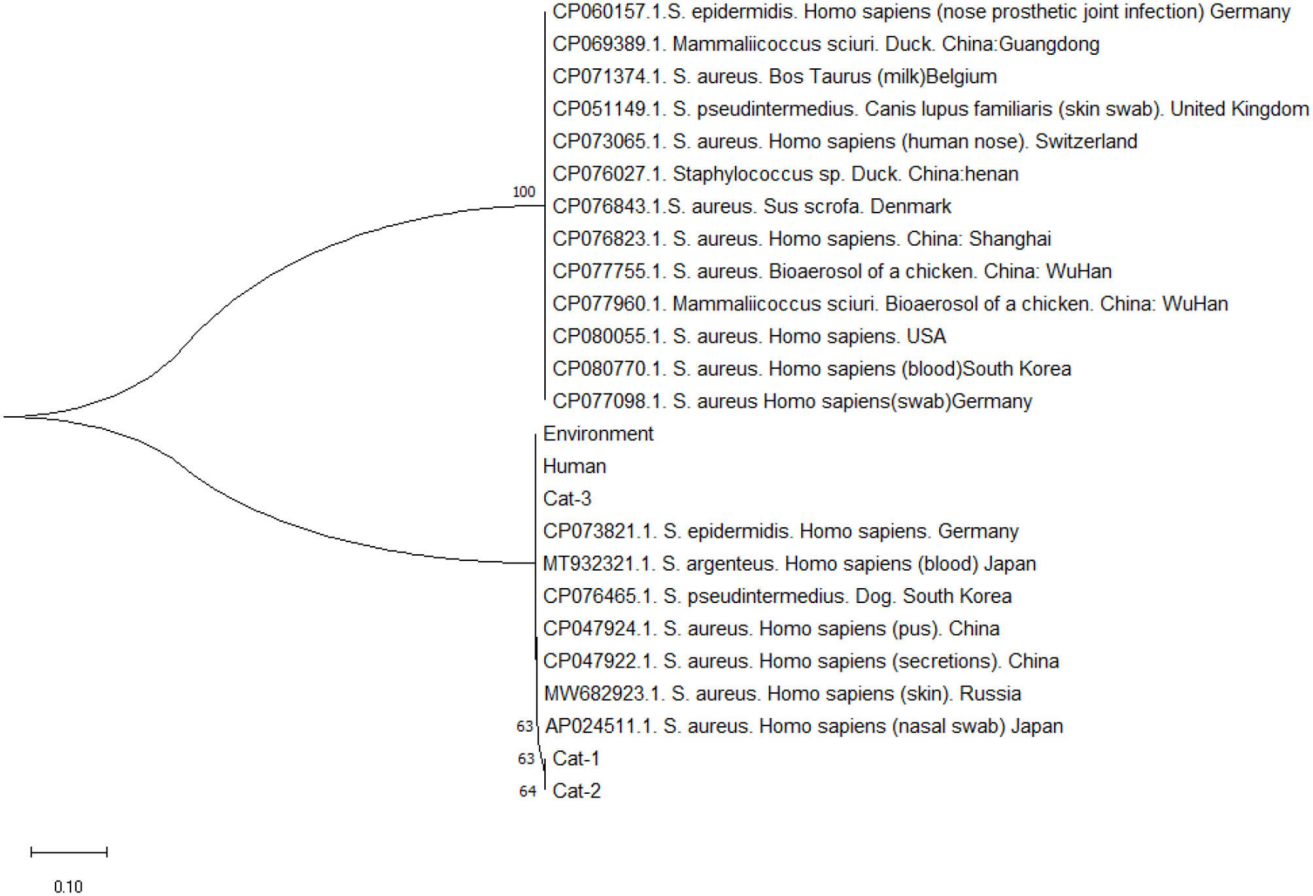
Phylogenetic tree of *Staphylococcus aureus mecA* gene.

Nucleic acid sequence alignment are given in Figure 3, and protein sequences alignment are given in Figure 4. One SNP was identified at position c.253C>A (Transvertion; Table 3). Cat-1 and Cat-2 sequences were 100% identical, while Cat-3, environmental and human sequences, were 100% identical. The phylogenetic tree (two clades) of *S. aureus mecA* gene was observed. *Staphylococcus aureus mecA* genes of cat, environment, and human samples were compared with NCBI database sequences. Local isolates of *S. aureus mecA* (Cat-1 and Cat-2) gene sequences were closely related to the *S. aureus mecA* gene isolated from *Homo sapiens* (nasal swab) from Japan. Environmental and human *mecA* gene sequences were closely related to *Staphylococcus epidermidis*. All samples (Cat-1, Cat-2, Cat-3, Environmental, and Human) cluster together with *S. aureus* from *H. sapiens* (pus, secretions, skin and nasal swab), *Staphylococcus argenteus* from *H. sapiens, Staphylococcus pseudintermedius* (dog), and *Staphylococcus epidermidis* (*H. sapiens*; Figure 5).

**Table 3:** Polymorphic sites in the *Staphylococcus aureus mecA* gene.

**Nucleotide position**	**Position 253**
Cat-1	C
Cat-2	C
Cat-3	A
Environmental	A
Human	A

**Figure 6:**
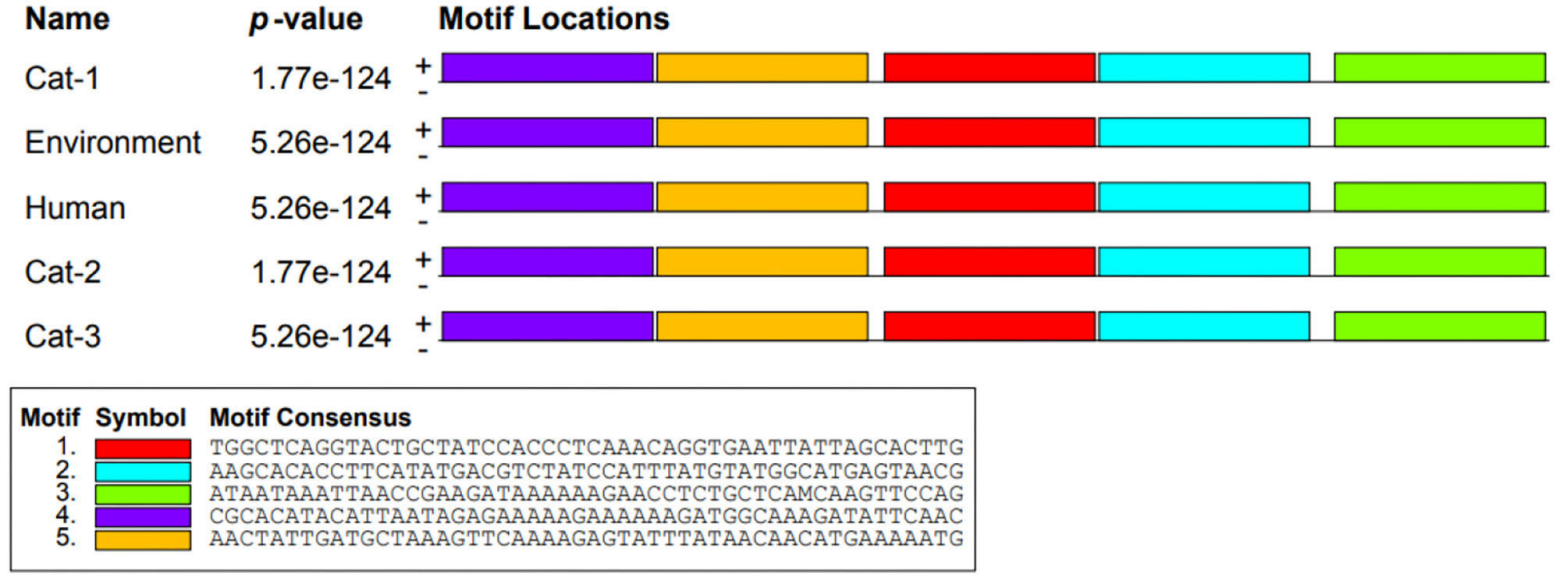
Nucleotide motifs of *Staphylococcus aureus mecA* gene.

**Figure 7:**
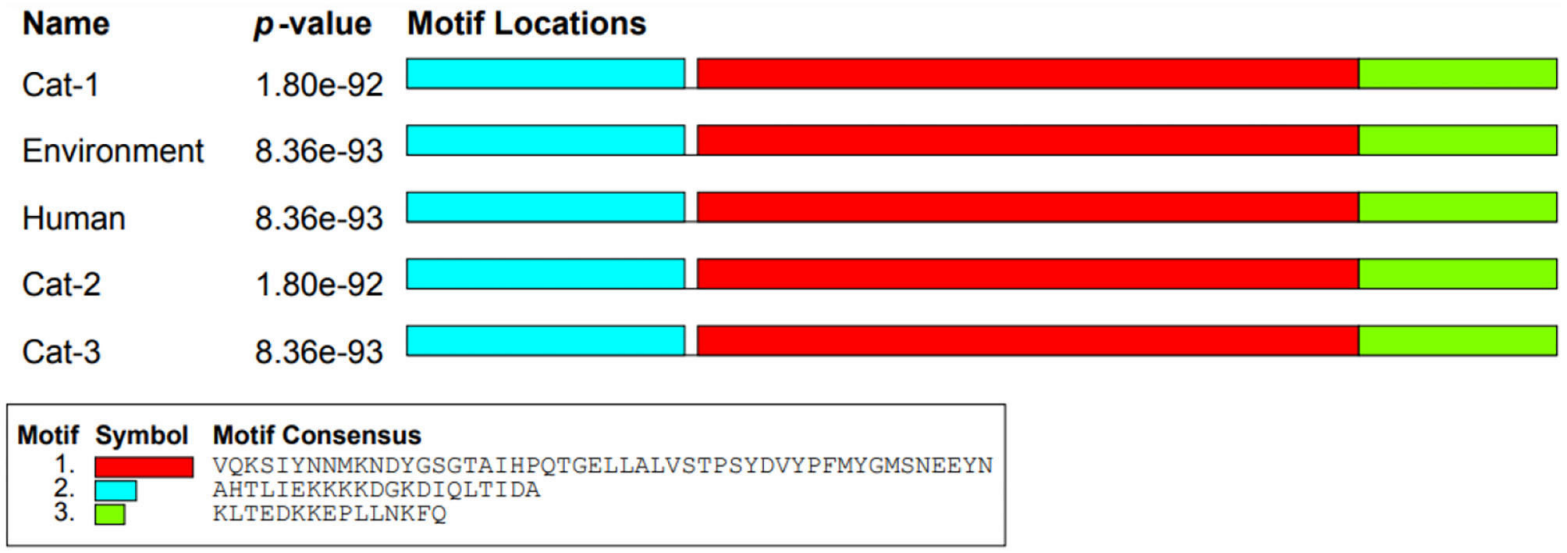
*mecA* protein motifs of *Staphylococcus aureus*.

**Figure 8:**
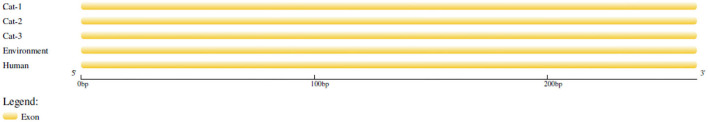
Gene structure of *Staphylococcus aureus mecA* gene amplified fragment.

Nucleic acid motifs (1,320 bp sequence) of Cat-1 and Cat-2 sequences *P*-value were found to be the same (1.77e-124), while Cat-3, environmental, and human sequences share same *P*-value (5.26e-124; Figure 6). The *P*-values of protein motifs (435 bp sequence) of Cat-1 and Cat-2 sequences were also found to be the same (1.80e-92). Similarly, Cat-3, environmental, and human sequences share same *P*-value (8.36e-93; Figure 7). Frequency of adenine and thymine nucleotide in motifs (0.334) and that of cytosine and guanine were the same (0.166). The coding region in yellow color was involved in the nucleotide structure (Figure 8). Threonine was replaced by asparagine (p.T84D) in Cat-3, environmental, and human samples (Table 4).

**Table 4:** Amino acid change in *Staphylococcus aureus mecA* protein.

**Amino acid position**	**Cat-1**	**Cat-2**	**Cat-3**	**Environmental**	**Human**
84	Threonine	Threonine	Asparagine	Asparagine	Asparagine

**Figure 9:**
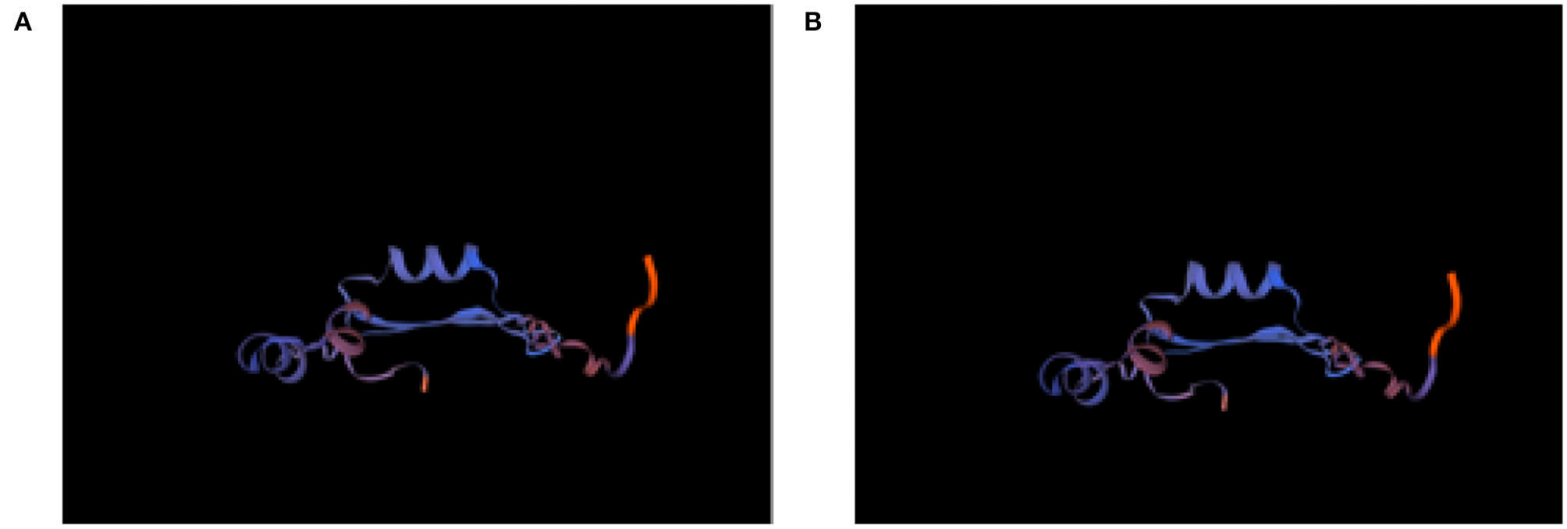
Protein structure of *Staphylococcus aureus mecA* gene **(A)** Cat-1 and Cat-2, and **(B)** Environment, Human, and Cat-3.

The protein structure of Cat-1 and Cat-2 proteins is found to be identical while Cat-3, environmental, and human proteins share identical structures (Figure 9). Physical and chemical properties of proteins Cat-1 and Cat-2 were found to be identical, while Cat-3, environmental, and human proteins have same physical and chemical properties (Table 5).

**Table 5:** *Staphylococcus aureus mecA* protein physical and chemical properties of proteins (Cat-1. Cat-2, Cat-3, Environmental, and Human).

**Sample ID**	**Cat-1 protein**	**Cat-2 protein**	**Cat-3 protein**	**Environmental protein**	**Human protein**
Number of amino acids	87	87	87	87	87
MW	9,915.28	9,915.28	9,928.28	9,928.28	9,928.28
pI	6.97	6.97	6.97	6.97	6.97
Number of negatively charged residues	12	12	12	12	12
Number of positively charged residues	12	12	12	12	12
Total number of atoms	1,397	1,397	1,397	1,397	1,397
II	36.30	36.30	39.03	39.03	39.03
Aliphatic index	72.87	72.87	72.87	72.87	72.87
GRAVY	−0.779	−0.779	−0.811	−0.811	−0.811

## Discussion

The human–animal bond has changed over the time. For example, the job of pets has shifted from working animals, e.g., catching mice and protecting houses, to animals that play roles as social pets, providing companionship. Pets may be important for their owners because they provide physical and mental health support but can also transmit number of zoonotic diseases (18). *Staphylococcus aureus* is one of the zoonotic pathogens that colonizes in the nasal cavity of pets and humans. *Staphylococcus aureus* is an infectious pathogen that acquires the ability to horizontally transfer genes between humans and animals (19). *Staphylococcus aureus* is recognized as major pathogen that causes major health problems in a community and is characterized by high rate of treatment failure worldwide (20). The One Health program is a global initiative plan for enhancing collaborations in every aspect of health care that involves humans, animals, and the environment. However, the role of dogs and cats in One Health communication is often underestimated. Therefore, the present study was conducted to investigate epidemiology and genetic relatedness of *S. aureus* by targeting the *mecA* gene at pet, pet owner, and environment interface. Nowadays, pets have become the major source of transmission of this pathogen to other animals and humans (21). In the last few years, much consideration has been paid on pets due to them serving as reservoir of antibiotic resistant bacteria and potential source of transfer of resistant genes from pets to humans (22). A study was conducted in Spain which reported co-carriage of ST398 strain of *S. aureus* in dogs and dog owners (23). Meanwhile, another study reported indistinguishable MRSA strains in humans and pets, suggesting the interspecies transmission of MRSA and *S. aureus* strains (24). Habibullah et al. (25) reported *S. aureus* that the prevalence in dogs (42.62%) and cats (37.50%) is twice higher than the current study. The prevalence of reported *S. aureus* contamination and/or infection in pet care workers is about six times less compared to current study findings (10% vs. 65.0%) (26). The high prevalence may be associated with unhygienic management practices in the study area, geographical differences, and unprotected interactions with pets (27). *Staphylococcus aureus* nasal colonization is associated with a number of factors, such as population type, geographical location, environmental factors, genetic factors, hormonal status, cell-wall composition, nasal secretions, antibiotic use, other infections, and immune level, which have been reported to be correlated with colonization (28). MRSA, increasingly being identified in both healthy and ill dogs and cats. It has been reported that *mecA* gene is the major evidence for the detection of MRSA isolates in Sudan (29). Consistent with current research, the results from two previous studies reported 46% and 49% MRSA in companion animals, respectively (30, 31). The relatively high prevalence in current studies and the high infection rate in hospitals and clinics are justified. High population density at clinics can cause the spread of pathogenic microorganisms into the environment.

Potential risks of carrying MRSA may include veterinary hospital staff, exposure to suppressant drugs, repeated utilization of the same antibiotics, use of non-specific antibiotics, and exposure to sources of infection carrying MRSA. MRSA can persist due to its antibiotic resistance ability, evolutionary adoption of biofilm covering, and its ability to bypass the immune system of hosts by utilizing specific molecular structures. Moreover, typical CA-MRSA, HA-MRSA, and LA-MRSA infecting non-specific hosts with MRSA strains is an additional indication for prolonged and persistent spread of MRSA strains (17). The high incidence of antibiotic resistance may be associated with the acquisition of resistance determinants (e.g., integrin's, plasmids, transposons, etc.) by vertical or horizontal gene transfer and, to a certain extent, with the misuse of antibiotics (5).

To curb the global spread of penicillin-resistant *S. aureus*, methicillin β-lactam antibiotics and subsequently oxacillin were manufactured. However, shortly after the use of methicillin, a MRSA emerged, making it one of the most life-threatening antibiotic-resistant pathogen (32). Methicillin resistance results from two distinct mechanisms. One is production of β-lactamases, which leads to a decrease in the antibiotic activity of β-lactams, and production of penicillin-binding protein 2a (PBP2a). PBP2a is an enzyme that is actively involved in the synthesis of peptidoglycan and promotes bacterial cell wall resistance. However, it does not have access to its active site, which binds to β-lactams, thereby interfering with its action and disrupting the normal process of bacterial cell wall synthesis (33). It is encoded by the mec gene, while the β-lactamase is encoded by the blaZ gene. The origin of the *mecA* gene is unknown. However, in some studies, the homologs of the *mecA* gene are found in *S. sciuri, S. lentus*, and *S. vitulinus* species. Since then, it has been hypothesized that these resistance determinants are derived from certain coagulase-negative staphylococci (CoNs). The species vitulinus suggests that this group may be the evolutionary ancestor of mecA. The mec genes are contained in the staphylococcal chromosome cassette mec (SCCmec), which is a mobile genetic component of staphylococci (34).

Another study conducted by Neamah et al. (1) found that *mecA* sequences from two clinically isolated cattle and human strains belong to the same group. Furthermore, another study conducted by Rolo et al. (35) studied the distribution pattern of the *mecA* gene, and showed that the earliest *Staphylococcus* species, including *S. sciuri, S. vitulinus*, and *S. fleurettii*, had an inclusive role in the stepwise aggregation of *SCCmec* elements and subsequently transferred to *S. aureus*. John et al. (36) conducted research to study 4,562 protein families and 1,764 unique genes of *S. aureus* and found 47 protein families that belonged to genes encoded by *SCCmec*, which include *mecA* gene, regulation genes (*mecR* and *mecI*), recombinase gene (ccrABC), transposons, insertion elements, heavy metals, and drug-resistant genes. Of the 152 *S. aureus* isolates, 73% were drug resistant and positive for *mecA* gene, while the remaining isolates were categorized as sensitive ones. Unsurprisingly, *mecA* was noted to be absent in most of the exposed strains, but, surprisingly, in scientist's dataset, 10% of sensitive *S. aureus* strains were composed of *SCCmec* elements that do not contain the *mecA* gene. The findings suggest that the presence of *SCCmec* element is not limited to only resistant strains of *S. aureus*.

The genomic composition of the core *SCCmec* gene, *mecA-mecR-mecI* was found in resistant strains of *S. aureus* and some species of *Staphylococcaceae*, such as *S. pseudointermedius, S. epidermidis, S. sciuri, S. argenteus, S. schleiferi, S. haemolyticus*, and *M. caseolyticus*, with mec-box genes found at 51 kb genes. The phylogenetic analysis based on the 16S rRNA gene sequence is widely used to study the evolutionary relationship of microorganisms (37). Interestingly, our study highlights the importance of those isolates as potential zoonotic agents. Hence, there is need to control and prevent this transmission by adopting clean living habits of pets and humans. Furthermore, the results of current study recommend more studies on whole genome sequence analysis along with multi locus sequence type (ST) approach in the future analyses to rule out the ST of each strain and the probability of raising a novel ST from the strains under study.

## Conclusion

The study witnessed rise in MRSA from pets, pet owners, and the environment as threat for public and animal health. Phylogenetic tree of submitted sequences revealed significant relatedness of MRSAs of animals, humans, and environment to each other. Replacement of amino acids and variation in structural proteins of MRSAs of different sources are indicative of genetic shifts. In such scenarios, novel strains of MRSAs are expected to emerge with modified resistance to antibiotics if stern precautions are not adopted.

## Data Availability Statement

The original contributions presented in the study are included in the article/supplementary material, further inquiries can be directed to the corresponding authors.

## Ethics Statement

The studies involving human and animal participants were reviewed and approved by the faculty and advanced studies board before the start of work, while approval of the completed research notified CE/1701/M.Phil., 2019 dated 11/10/2019. Post study ethical permission certificate was also obtained vide FVS/379/26.02.2020. The patients/participants provided their written informed consent to participate in this study. Written informed consent was obtained from the owners for the participation of their animals in this study.

## Author Contributions

MS did research and wrote the original manuscript draft. AA, MI, and WP contributed in project design and check the validity of the research. MA and HS executed the bioinformatics analysis. AG and MSH helped in laboratory work. ZB and MK did review and editing of manuscript. KA helped in clinical data collection. All authors contributed to the article and approved the submitted version.

## Funding

This work was supported by the Agricultural Science and Technology Innovation Program of the Chinese Academy of Agricultural Sciences (25-LZIHPS-03).

## Conflict of Interest

The authors declare that the research was conducted in the absence of any commercial or financial relationships that could be construed as a potential conflict of interest.

## Publisher's Note

All claims expressed in this article are solely those of the authors and do not necessarily represent those of their affiliated organizations, or those of the publisher, the editors and the reviewers. Any product that may be evaluated in this article, or claim that may be made by its manufacturer, is not guaranteed or endorsed by the publisher.

## Abbreviations

*S. aureus*: *Staphylococcus aureus*
MRSA: Methicillin-resistant *Staphylococcus aureus*
HGT: horizontal gene transfer
ARG: antibiotic resistance genes
VFG: virulence factors-encoding genes
LA-MRSA: livestock-associated MRSA
HA-MRSA: healthcare-associated MRSA
CA-MRSA: community-associated MRSA
SCCmec: Staphylococcal Chromosomal Cassette mec
MIC: minimum inhibitory concentration
NCBI: National Center for Biotechnology Information
ST: sequence type.
